# Species-specific effects of microplastics on juvenile fishes

**DOI:** 10.3389/fphys.2023.1256005

**Published:** 2023-08-04

**Authors:** Chaonan Zhang, Fei Wang, Qiujie Wang, Jixing Zou, Junjie Zhu

**Affiliations:** ^1^ Department of Environmental Science, Zhejiang University, Hangzhou, China; ^2^ National-Local Joint Engineering Laboratory of Aquatic Animal Genetic Breeding and Nutrition, Zhejiang Provincial Key Laboratory of Aquatic Resources Conservation and Development, College of Life Science, Huzhou University, Huzhou, China; ^3^ Joint Laboratory of Guangdong Province and Hong Kong Region on Marine Bioresource Conservation and Exploitation, College of Marine Sciences, South China Agricultural University, Guangzhou, China

**Keywords:** microplastics, species-specific, juvenile fish, gene expression, intestinal morphology

## Abstract

Microplastics contamination have been extensively reported in aquatic ecosystem and organisms. It is wildly acknowledged that the ingestion, accumulation and elimination of microplastics in fishes are species-specific, which mainly depending on the feeding behavior. This study aimed to investigate the effects of microplastics on the morphology and inflammatory response in intestines of fishes with different feeding types. Largemouth bass (carnivorous fish), grass carp (herbivorous fish) and Jian carp (omnivorous fish) were used as organism model. The contributing concentration and size of microplastics were explored as well as the response time and legacy effect in fishes. Two different sizes of polystyrene microplastics (80 nm and 8 μm) were set at three concentrations. And samples were analyzed at different exposure times and depuration times. Histological analysis indicated that multiple abnormalities in intestines were presented in three species fishes after acute exposure microplastics. The mRNA abundance of immune-related genes in the intestine tissues of fishes were significantly fluctuant. There were differential expressions of genes coping with differential sizes and concentrations of microplastics exposure in different fishes. The reason for the difference effects of microplastics on fishes was still unclear but could be due to the difference in the structure and function of the digestive system. These results provided a theoretical basis to further analysis of the mechanism of fish intestinal pathology caused by microplastics.

## 1 Introduction

Plastics have been remarkable materials in peoples’ daily life due to its versatile, durable, and incredibly adaptable. Plastics production reached 390 million tonnes in 2021 worldwide with approximately 9% increasing rate every year and China contributed to 32% of world’s plastics production (Plastics Europe, 2022). In the meanwhile, the global total of plastic waste reached 380 Tg in 2018 with an exponential growth every year (Rai et al., 2021). Once entering the environment, plastic would degrade or fragment into microplastics through UV radiation, mechanical transformation or biological degradation by microorganisms (Cole et al., 2011; Alimi et al., 2018). Microplastics are defined as small plastic pieces or fibers smaller than 5 mm (NOAA, 2015). They come in many forms, not only secondary sources, but also primary sources, such as microbeads in personal care products (McDevitt et al., 2017). Microplastics contamination have been extensively reported in marine, freshwater and terrestrial ecosystems (Wang et al., 2020a; Peng et al., 2020; Xu et al., 2020), thus identified as one of the top 10 emerging global environmental problems by the United Nations Environment Program.

Adverse effects of microplastics on fishes have been found in many literatures (Jacob et al., 2020; Anna et al., 2021; Mallik et al., 2021). Due to the attractive color, buoyancy, and food-like properties, fish are particularly prone to ingesting microplastics (Garrido Gamarro et al., 2020). The ingestion of microplastics by fish can cause a variety of consequences: 1) microplastics can lead to physical damage and histopathological alterations (Peda et al., 2016; Jabeen et al., 2018; Ahrendt et al., 2020); 2) microplastics can cause impairments in oxidative, and disorders of inflammatory balance and intestinal microflora (Gu et al., 2020; Huang et al., 2020; Iheanacho and Odo, 2020); 3) microplastics can also lead to fish behavior changes (Brun et al., 2019; Guimarães et al., 2021; Rios-Fuster et al., 2021; Shi et al., 2021); 4) microplastics can act as carriers to intensify further adverse effects of other pollutants on fish (Banaee et al., 2019; Zhang et al., 2019; Li et al., 2023a).

It is wildly acknowledged that the ingestion, accumulation and elimination of microplastics in fishes are species-specific (Mizraji et al., 2017; Xu and Li, 2021). The field investigation found microplastic amounts in filter-feeding and omnivorous fish were higher than that of carnivorous species (Wang et al., 2020b). The laboratory experiment proved that microplastics ingestion in fish larvae was influenced by feeding type of fish, and omnivores fish were less able to eliminate microplastics than filter-feeding fish (Zhang et al., 2021). However, the physiological effects of micro-nano plastics on juvenile fish with different feeding habits have not been reported.

In this study, species-specific effects of microplastics on three commercial fish species with different feeding types were investigated. Largemouth bass, *Micropterus salmoides* is a typical freshwater carnivorous fish species and widely farmed in China due to its strong adaptability, fast growth, delicious taste, and high economic value (Wang et al., 2020c). Grass carp (*Ctenopharyngodon idella*), a herbivorous fish species, is one of the most important freshwater cultivars in China, which annual production exceeded 5.53 million tons in 2019 (China Fishery Statistical Yearbook, 2020). Jian carp (*Cyprinus carpio* var. Jian) is an omnivorous freshwater fish species with an annual production of 24.2 million tons worldwide (Lin et al., 2019; Li et al., 2023b). This study aimed to reveal the effects of microplastics on the morphology and inflammatory response in intestines of fishes with different feeding types. To achieve this goal, histopathological sections were examined, and immune-related genes profiles were used to study the changes in the intestinal tissue of three fishes after microplastics exposure. These results would provide a theoretical basis to further analysis of the mechanism of fish intestinal pathology caused by microplastics.

## 2 Material and method

### 2.1 Materials

Polystyrene microplastics with diameters of 80 nm and 8 μm were purchased from Dae Technology Company (Tianjin, China). Largemouth bass, grass carp and Jian carp were bought from a livestock farm in Shunde City (Guangdong, China). Largemouth bass was (5.23 ± 0.62) cm in length and (2.97 ± 0.64) g in weight. Grass carp was (5.81 ± 0.50) cm in length and (3.82 ± 0.91) g in weight. Jian carp was (3.46 ± 0.16) cm in length and (0.93 ± 0.19) g in weight. Fish were acclimatized at 25.2 ± 1.5 °C in culture water (pH 7.1 ± 0.4; dissolved oxygen 6.4 ± 0.5 mg/L) with a 12 h light/dark cycle. Before the experiment, fish were acclimated in 100 L glass tanks for 5 d and were fed with 5.0% body weight fodder (Haid Group, Guangdong, China) twice daily.

### 2.2 Experimental design

Two different sizes of fluorescent microplastics (80 nm and 8 μm) were set at four concentrations for grass carp and Jian carp: 0, 0.02 mg/L, 0.2 mg/L and 2 mg/L. Based on the previous findings (Zhang et al., 2021), carnivorous fish seemed to be more tolerant to microplastics than other fishes. So, the higher microplastics exposure concentrations (0.05 mg/L, 0.5 mg/L and 5 mg/L) for largemouth bass were set. The concentrations of exposure for MPs were selected based on the other studies (Ding et al., 2018; Li et al., 2020; Zhang et al., 2021). The microplastics with nanometer particle size (80 nm) and micron particle size (8 μm) were compared.

In the exposure experiment, tanks (20 cm × 15 cm × 15 cm) were filled with 2 L of culture water and eight fish. A total of twenty-four tanks were set for each fish species, including control group and replicate group. Each species of fish was tested separately. Three replicate tanks were used for 24 h and 48 h sampling times. After 48 h exposure, the surviving fish were moved to an aquarium with clean water containing no microplastics for 48 h. No feeding was done during exposure and depuration. At 24 and 48 h after exposure and clearance, two fish were dissected from each tank and the intestines were removed for subsequent analysis. This study was carried out in strict accordance with the recommendations in the Guide for the Care and Use of Laboratory Animals of the National Institutes of Health. All surgery was performed under anesthesia, and all efforts were made to minimize suffering.

### 2.3 Histopathological analysis

A total of 24 fish from the control and experimental groups were anesthetized on ice and intestines were dissected. Intestinal tissue fixed in general-purpose tissue fixator (Servicebio, Wuhan, China), embedded in paraffin wax, sectioned at 4 μm thickness, and stained with hematoxylin-eosin (H&E). Tissue slices were examined and photographed by a microscopy (Nikon, Tokyo, Japan) with the Mshot Image Analysis System.

### 2.4 RNA extraction and cDNA synthesis

The experimental methods of RNA extraction and cDNA synthesis are presented in Supplementary Text S1. The cDNA was stored at −80°C until further analysis.

### 2.5 Immune and enzyme-related gene expression

The SYBR green real-time PCR assay was performed on the CFX Connect TM Real-Time System (BIO-RAD, Hercules, CA, USA) using the SYBR^®^ Green Premix Pro Taq HS qPCR kit (Accurate Biotechnology Co., Ltd., Hunan, China) following the manufacturer’s approach. Specific primer sequences are listed in Table 1. Details of the PCR program are presented in Supplementary Text S2. Expression levels of target genes were normalized to the internal reference, and the data were calculated as the fold change in comparison to the control group.

**TABLE 1:** List of gene primers used for qPCR.

Fish	Genes	Sequence, forward/reverse (5′–3′)
Largemouth bass	*β-actin*	F: ATC​GCC​GCA​CTG​GTT​GTT​GAC
		R: CCT​GTT​GGC​TTT​GGG​GTT​C
	*IL-8*	F: GAG​CCA​TTT​TTC​CTG​GTG​ACT
		R: TCC​TCA​TTG​GTG​CTG​AAA​GAT​C
	*Caspase 3*	F: GCT​TCA​TTC​GTC​TGT​GTT​C
		R: CGA​AAA​AGT​GAT​GTG​AGG​TA
Grass carp	*β-actin*	F: GGCTGTGCTGTCCCTGTA
		R: TTA​TTG​TGG​TTA​CGC​TGG​A
	*IL-1β*	F: AGA​GTT​TGG​TGA​AGA​AGA​GG
		R: TTA​TTG​TGG​TTA​CGC​TGG​A
	*IL-8*	F: ATG​AGT​CTT​AGA​GGT​CTG​GGT
		R: ACA​GTG​AGG​GCT​AGG​AGG​G
	*TGF-β1*	F: TTGGGACTTGTGCTCTAT
		R: AGTTCTGCTGGGATGTTT
	*TNF-α*	F: CGCTGCTGTCTGCTTCAC
		R: CCTGGTCCTGGTTCACTC
Jian carp	*18S*	F: CTG​AGA​AAC​GGC​TAC​CAT​TC
		R: GCC​TCG​AAA​GAG​ACC​TGT​ATT​G
	*IL-1β*	F: GAG​TGA​ACT​GCA​CCA​AAC​AAC
		R: GTC​GGC​ACT​GTC​AGA​GTA​AAT
	*IL-10*	F: CTC​CGT​TCT​GCA​TAC​AGA​GAA​A
		R: TCA​TGA​CGT​GAC​AGC​CAT​AAG
	*TGF-β*	F: ACG​TTT​CCA​GAT​GGT​TCA​GAG
		R: GCC​ACT​TTC​TTT​GTT​TGG​GAA​TA
	*TLR-2*	F: GTG​CTC​CTG​TGA​GTT​TGT​ATC​T
		R: TGG​AGT​GTC​GCA​CAC​ATA​ATA​G

### 2.6 Statistical analysis

All data were quantified as the mean ± standard error (S.E) and performed by one-way ANOVA using SPSS 17.0 and Excel 2016. Statistical significance between the control and the experimental groups was conducted by the Duncan’s multiple range test. A value of *p* < 0.05 was set with statistical significance.

## 3 Results

### 3.1 Intestinal morphology

**FIGURE 1:**
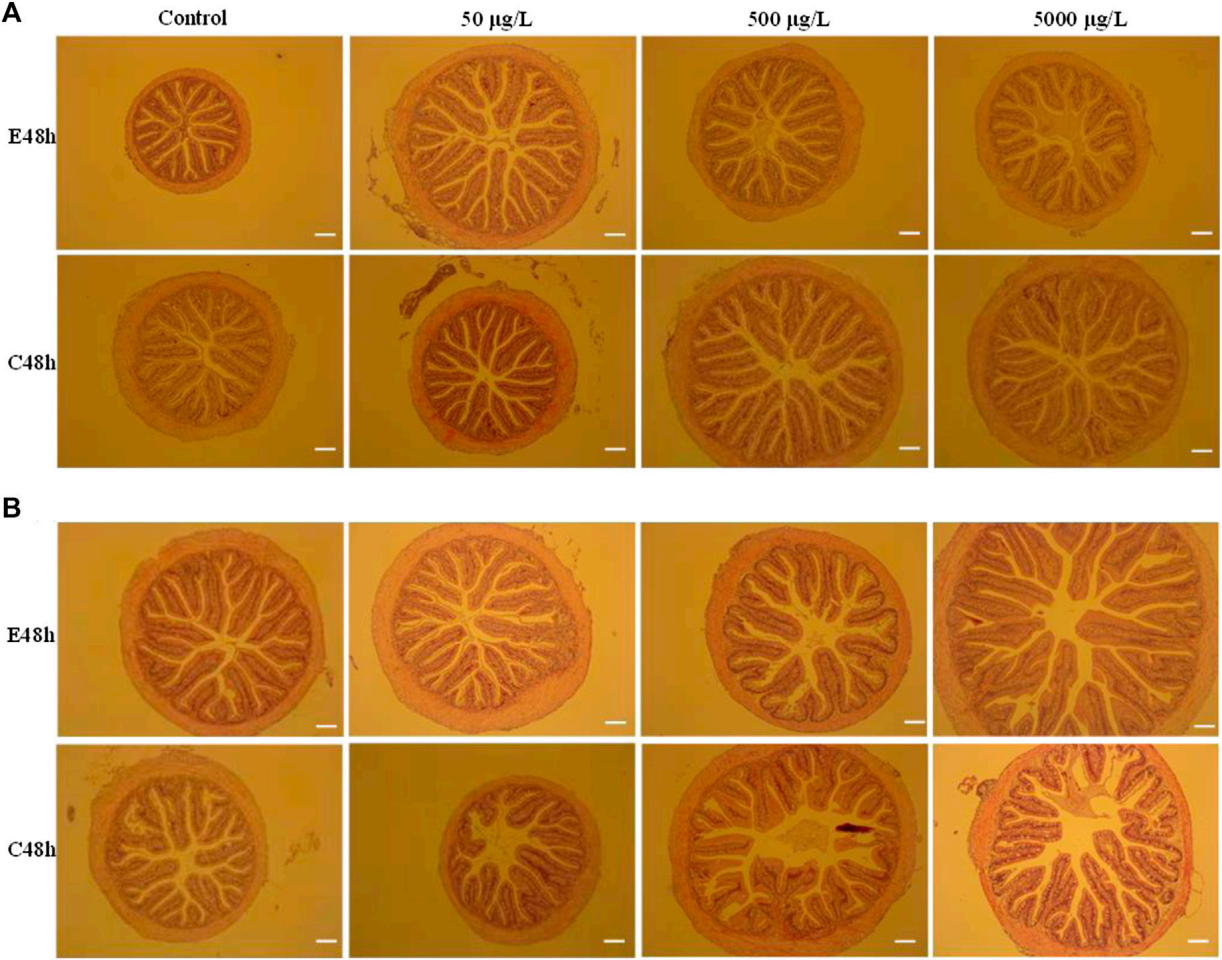
Histopathological analysis of intestines of largemouth bass juvenile exposed to polystyrene microspheres of 80 nm **(A)** and 8 μm **(B)** after exposure 48 h and clean 48 h. Exposure concentration and time were shown in the picture. Scale bar = 20 μm.

After HE staining, intestinal histomorphology of three fishes were examined using a light microscope. Histopathological sections showed that the intestinal folds of largemouth bass juvenile were in disorder and shortened, infiltrated cells, especially when fish were exposure in higher concentration microplastics of micron scale (Figure 1). In the intestine of grass carp juvenile, there was no difference between the control and the treatments for vacuolization, goblet cell hyperplasia or villus shortening (Supplementary Figure S1). After combing all the scored histopathology features together, there was no significant difference in the intestinal muscular thickness and intestinal villi length between the groups (*p* > 0.05) (Supplementary Figure S2). Juvenile Jian carp showed multiple abnormal intestines after microplastics exposure (Supplementary Figure S3). The intestinal folds in the experimental group were not full or regular. However, no significant difference was found in the muscle thickness or villi length in Jian carp either (*p* > 0.05). Histopathological data of Jian carp are listed in Supplementary Table S1.

### 3.2 Transcriptional responses of target genes

**FIGURE 2:**
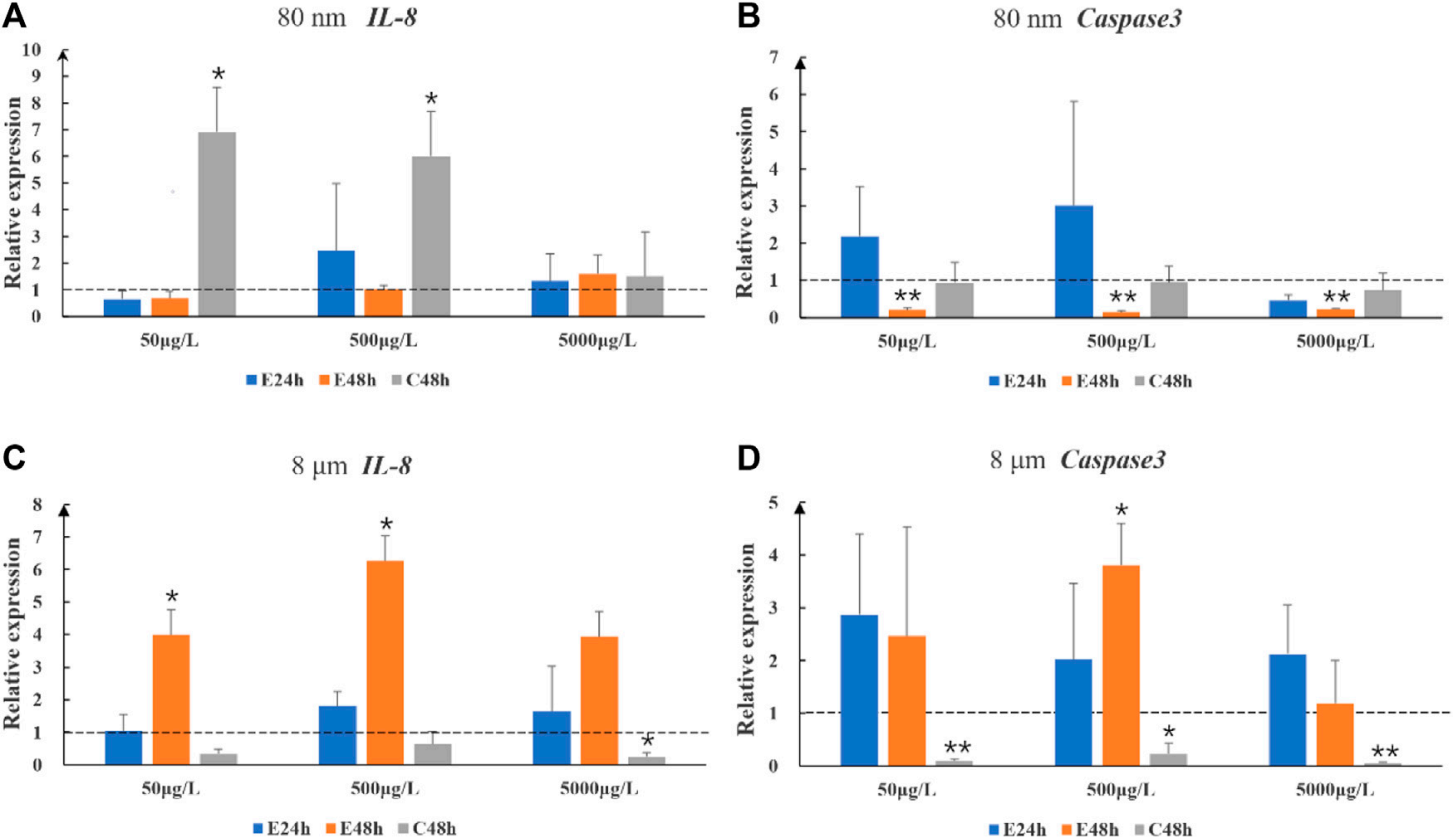
The relative gene expression levels (fold change) of *IL-8* **(A,C)** and *Caspase 3* **(B,D)** in intestines of largemouth bass juvenile exposed to microplastics. Data are expressed as mean ± standard deviation. Significant differences from control are shown (*p < 0.05; **p < 0.01).

After 8 μm microplastics exposure 48 h, the expression levels of the immune-related gene (*IL-8*) were significantly upregulated in the intestines of largemouth bass juvenile (*p* < 0.05) (Figure 2C). 80 nm microplastics caused upregulation of *IL-8* in 48 h depuration after exposure 48 h (Figure 2A). Whereas the situation of high concentration exposure was different with mid and low concentration exposure (Figures 2A, C). Expression of *Caspase 3* gene in the intestines of fish exposed 80 nm microplastics 48 h and cleaned 8 μm microplastics 48 h were significantly lower than that in the intestines of fish in the control group (*p* < 0.01) (Figures 2B, D).

**FIGURE 3:**
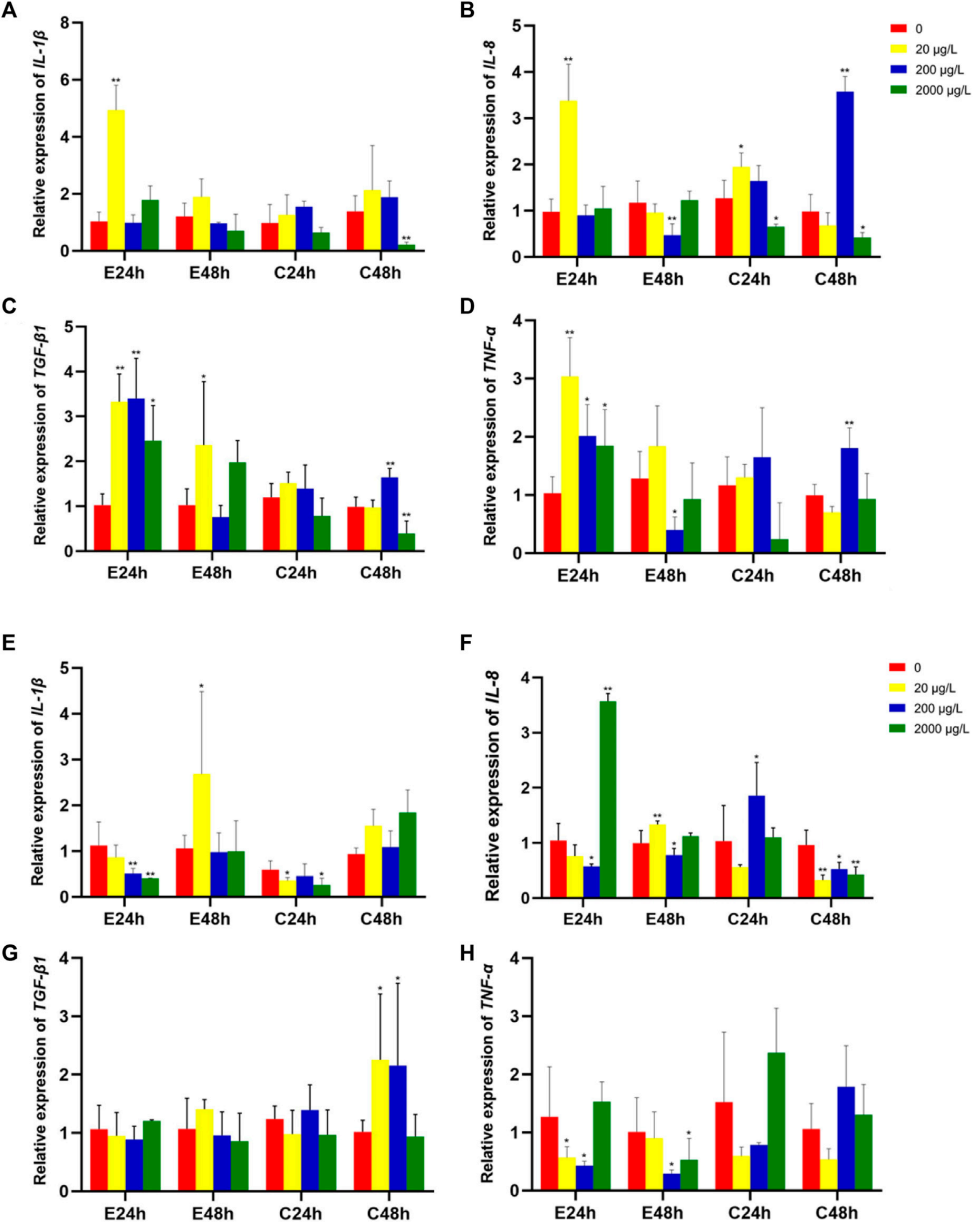
The relative gene expression levels (fold change) in intestines of grass carp juvenile exposed to microplastics of 80 nm **(A–D)** and 8 μm **(E–H)**. Data are expressed as mean ± standard deviation. Significant differences from control are shown (*p < 0.05; **p < 0.01).

The effects of microplastics on the expression of levels of immune-related genes in intestine tissues of grass carp are shown in Figure 3. The relative expression levels of *IL-1β*, *IL-8*, *TGF-β1* and *TNF-α* were all observably upregulated (*p* < 0.01) when exposure 80 nm microplastics at low concentration (20 μg/L) in the start of 24 h. *TGF-β1* and *TNF-α* expression level when exposure 80 nm microplastics 24 h at middle and high concentration (200 μg/L and 2000 μg/L) were significantly upregulated, rather than *IL-1β* and *IL-8* expression level. However, there was different gene expression pattern when exposure 8 μm microplastics.

**FIGURE 4:**
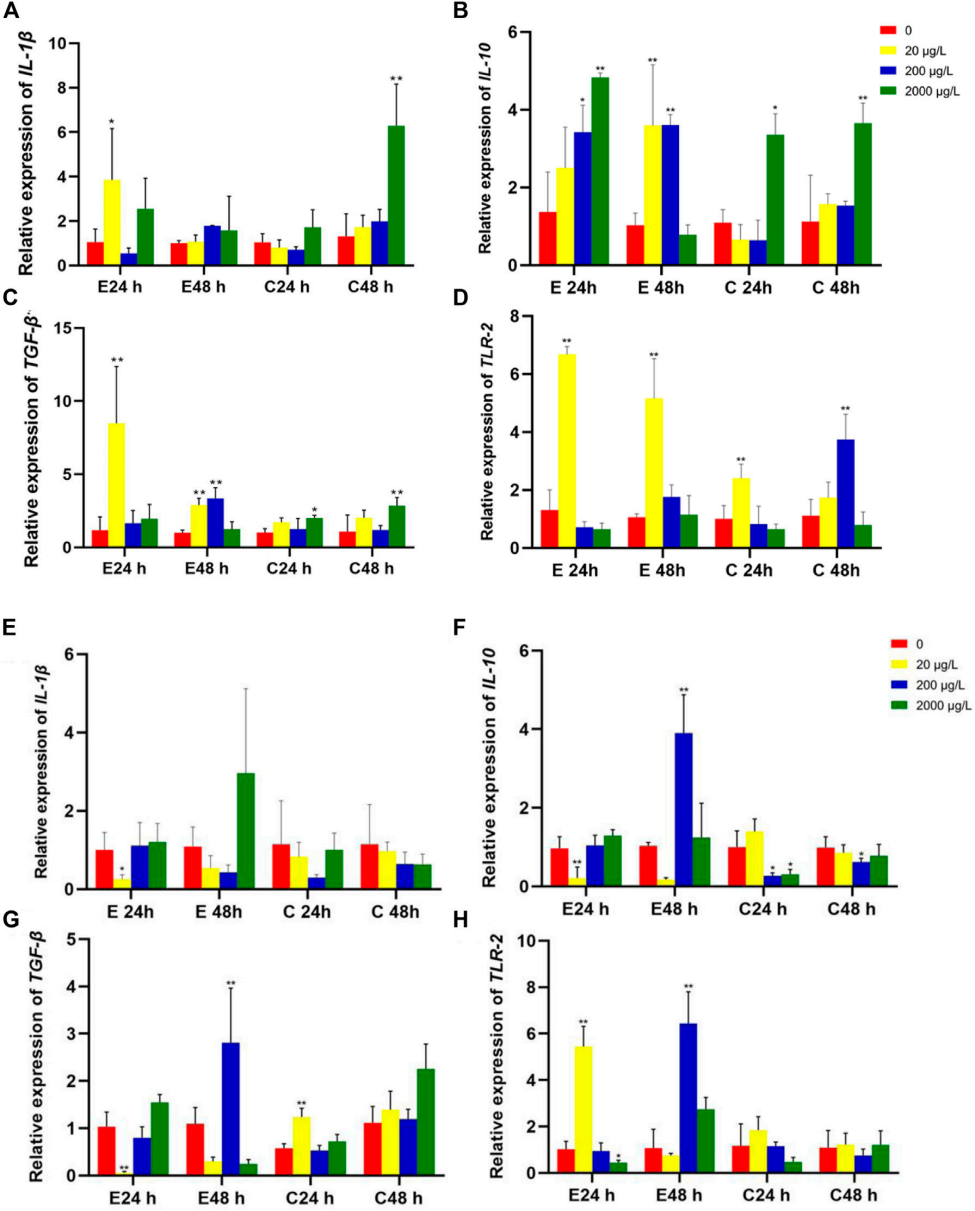
The relative gene expression levels (fold change) in intestines of Jian carp juvenile exposed to microplastics of 80 nm **(A–D)** and 8 μm **(E–H)**. Data are expressed as mean ± standard deviation. Significant differences from control are shown (*p < 0.05; **p < 0.01).

The mRNA expression levels of *IL-1β*, *IL-10*, *TGF-β* and *TLR-2* in intestines of Jian carp juvenile exposed to microplastics of 80 nm and 8 μm are shown in Figure 4. The upregulation of pro-inflammatory cytokines, such as *IL-1β* and *TLR-2*, or/and downregulation of anti-inflammatory cytokines including *TGF-β1* and *IL-10* could cause inflammation in fish. Noteworthily, Jian carp cured better in 8 μm microplastics treatment than in 80 nm microplastics treatment.

## 4 Discussion

### 4.1 Effects of microplastics on intestinal morphology of fish

The intestinal morphological effects of microplastics with a dose-dependent way have been explored in various fishes. Over secretion of goblet cells was found in juvenile guppy (*Poecilia reticulata*) after exposing microplastics with 32–40 μm diameter, and the higher concentration of microplastics, the more goblet cells secreted (Huang et al., 2020). However, the loss of villus and crypt cells was significantly increased due to microplastic physical abrasion in the intestine of juvenile intertidal fish (*Girella laevifrons*), and leukocyte infiltration and hyperemia exposure in the high concentration group were more serious than those in the low concentration group (Ahrendt et al., 2020). In the European sea bass (*Dicentrarchus labrax* L.), intestinal tissues were altered after fish were fed with polyvinyl chloride (PVC) pellets for 90 days (Peda et al., 2016). Another morphometric analyses of sea bass fed polyethylene (PE) microplastics in the diets for 21 days showed a significant reduction in the amounts of goblet cells as well as a decrease in villus height (Espinosa et al., 2019). Histological analysis indicated that multiple abnormalities in intestines are presented in three species fishes after acute exposure microplastics in this study.

As we all known, intestine is vital for the digestion and absorption of nutrients, and intestinal morphology characters, such as muscular layer thickness, villi length, and the number of goblet cells indicate intestine health in fish. To some extent, abnormal in the intestinal sections is an immune response to external stimulus. On one hand, pathological changes of intestinal tract might be the result of microplastics intrusion. On the other hand, it is crucial to determine whether this intrusion outpaces the organism’s ability to repair itself. From histopathological analysis of intestines of largemouth bass juvenile exposed to 8 nm and 8 μm microspheres after exposure 48 h and clean 48 h (Figure 1), we found microplastics of lager size and higher concentration cause more serious damage, and the damage seems to be irreversible. Obviously, this change makes fish more sensitive to infection by pathogens. Compared with the intestinal slices of grass carp and Jian carp, Jian carp with smaller intestinal diameter and less perfect villus structure was more seriously damaged by microplastic invasion.

### 4.2 Effects of microplastics on immune-related genes expression of fish

Many animal studies have indicated that exposure to microplastics impairs oxidative and inflammatory bowel balance (Choi et al., 2018; Ding et al., 2018). Especially, microplastics cause intestinal inflammation, manifested by a significant increase in *IL-1α* levels in the intestine (Hirt and Body-Malapel, 2020). The immune function of organs is highly correlated with the inflammatory response, which is generally considered to be a typical defense response that protects the host from pathogens (Zhong et al., 2020). Cytokines mediate the inflammatory response in fish, which are mainly divided into pro-inflammatory factors (e.g., *TNF-α*, *IL-1β* and *IL-10*) and anti-inflammatory factors (e.g., *IL-10* and *TGF-β*). For example, interleukin is a typical class of cytokines which is mainly involved in regulating all kinds of lymphocytes in the immune system. Tumor necrosis factor α (*TNF-α*), as pleiotropic proinflammatory and potent regulatory cytokines, can regulate cell proliferation, apoptosis or differentiation in the immune system (Cao et al., 2020). Toll-like receptors (*TLRs*), as a crucial innate receptor, can identify pathogen-associated molecular patterns (PAMPs) of invading microorganisms and induce downstream *NF-κB* activation and the production of *TNF-α*, *IL-10* and other cytokines (Meng et al., 2021).

Previous research in adult male zebrafish (*Danio rerio*) showed that exposure to 1,000 μg/L of 0.5 μm microplastics for 14 days significantly upregulated the transcription levels of *IL-1α*, *IL-1β*, and *Ifn* in the intestine (Jin et al., 2018). In the present study, microplastics exposure significantly induced or restrained the mRNA expression of immune-related genes in the intestine tissues of fishes. There were differential expressions of genes coping with differential sizes and concentrations of microplastics in different fishes. Similarly, in other species, such as rats (Wei et al., 2021) and prawn (Li et al., 2023a/b), the mRNA abundance of immune-related genes was increased with microplastics exposure.

### 4.3 Response time and legacy effect of microplastics with different concentration and size

In terms of damage to intestinal morphology, acute exposure did not cause significant damage at the size and concentration of microplastics exposed in this paper. From the perspective of gene expression level, when exposed to nanoscale microplastics at low concentration, fish can promote self-repair through the upregulation of some inflammatory factors. For micron-scale microplastics, we hypothesized that part of microplastics could be removed by fish excretion after ingestion. Therefore, there was no significant difference in gene expression between the experimental fish and the control group during the recovery period. The effects of microplastics on juvenile fishes are species-specific, the specific mechanism needs to be further studied.

Although time had no significant effect on intestinal morphology, we hypothesized that it was related to exposure conditions. Thankfully, even when exposed to extremely high concentration (mg/L) of microplastics, there is no immediate visible damage to the intestinal morphology of fish. Response time and recovery time of gene expression was species-specific. Grass carp has the longest intestinal tract, followed by Jian carp, and largemouth bass has the shortest intestinal tract, which is related to their feeding habits. We hypothesize that the lag time of microplastics in fish intestine is related to the length of the intestine. A methodology to assess how effective Mediterranean fish species, that are known to have ingested marine plastic, were considered gut length as well, which showed fish with smaller gut length is more representative (Bray et al., 2019).

## 5 Conclusion

In this study, species-specific effects of microplastics on three fishes with different feeding types were investigated. The contributing concentration and size of microplastics, as well as the response time and legacy effect in fishes were also explored. Two different sizes of fluorescent microplastics (80 nm and 8 μm) were set at four concentrations. Multiple abnormalities in intestines were presented in three species fishes, and there were differential expressions of genes coping with differential sizes and concentrations of microplastics exposure in different fishes. The results of this study would be beneficial for extrapolating microplastics contamination risks to commercial fishes. The reason for the difference effects of microplastics on fishes was still unclear but could be due to the difference in the structure and function of the digestive system. This study will provide a valuable steppingstone for future research, where we hope to address the microplastics research gap between various fish species.

## Data Availability

The original contributions presented in the study are included in the article/Supplementary Materials, further inquiries can be directed to the corresponding author.
