# Supplementary material for: Species-specific effects of microplastics on juvenile fishes
Source: Front Physiol. 2023 Aug 4;14:1256005. doi: 10.3389/fphys.2023.1256005 (PMC10436232; doi:10.3389/fphys.2023.1256005)
Supplement: Supplementary file 1 [file DataSheet1.DOCX]

**Species-specific effects of microplastics on juvenile fishes**

Chaonan Zhang^1,2,3^, Fei Wang^2^, Qiujie Wang^3^, Jixing Zou^3^, Junjie Zhu^2^*

^1^Department of Environmental Science, Zhejiang University, Hangzhou 310000, China

^2^National-Local Joint Engineering Laboratory of Aquatic Animal Genetic Breeding and Nutrition, Zhejiang Provincial Key Laboratory of Aquatic Resources Conservation and Development, College of Life Science, Huzhou University, Huzhou 313000, China

^3^Joint Laboratory of Guangdong Province and Hong Kong Region on Marine Bioresource Conservation and Exploitation, College of Marine Sciences, South China Agricultural University, Guangzhou 510642, China

Correspondence author: * ^[*](mailto:*Zhjj@zjhu.edu.cn)^[Zhjj@zjhu.edu.cn](mailto:*Zhjj@zjhu.edu.cn) (Junjie Zhu)

Numbers of figures: 3

Numbers of tables: 1

Numbers of texts: 2

Numbers of pages: 7

Supplementary figure 1. Intestinal muscular thickness and intestinal villi length of grass carp.


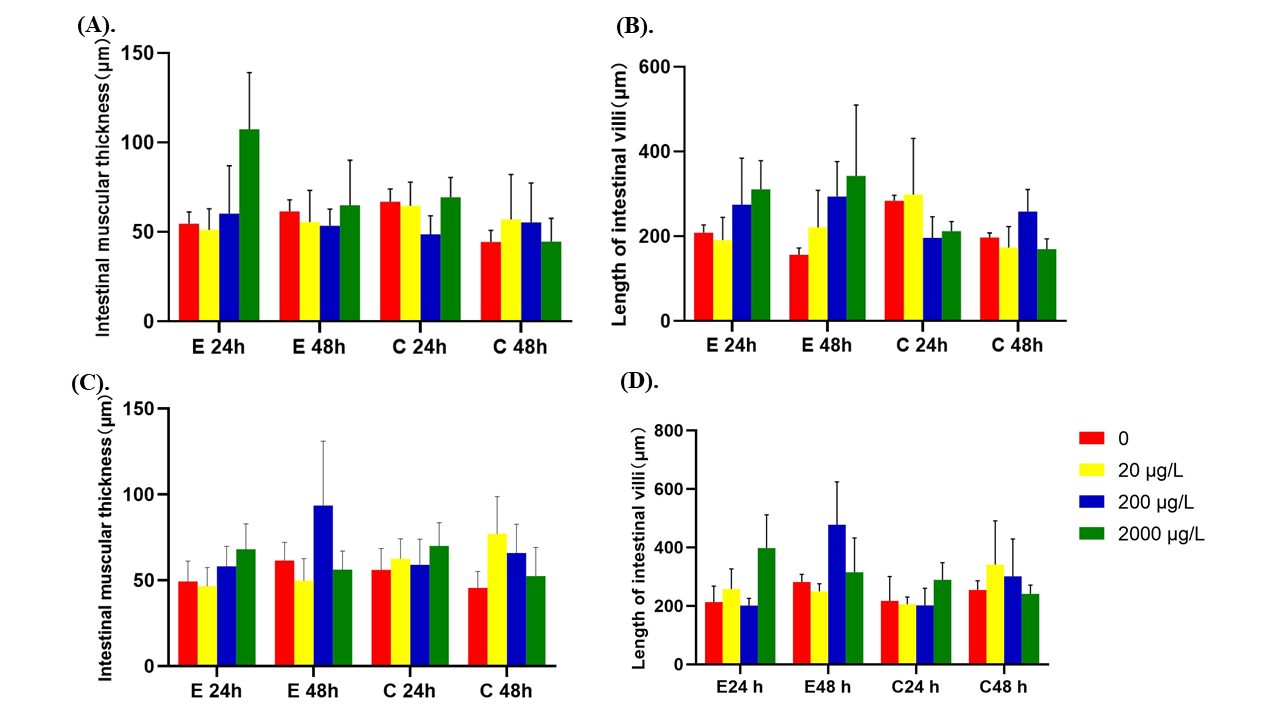


Supplementary figure 2. Histopathological analysis of intestines of grass carp juvenile exposed to polystyrene microspheres. Scale bar = 50 μm.


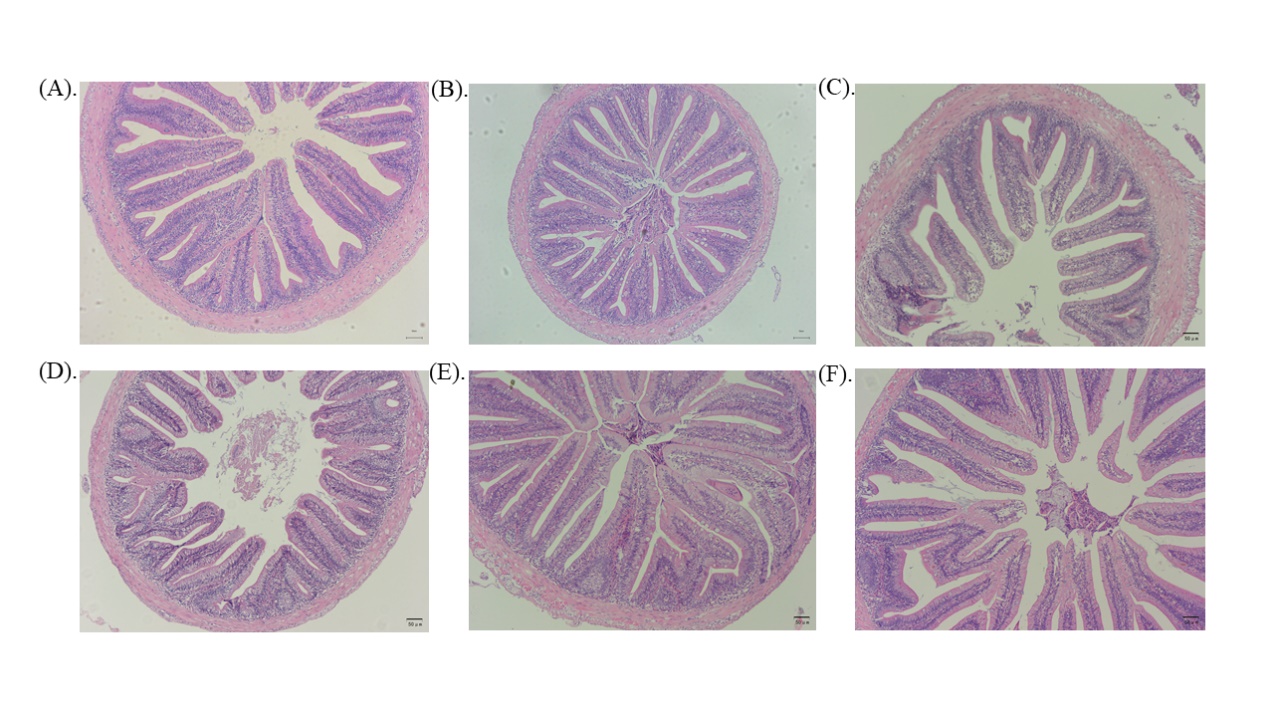


Supplementary figure 3 Histopathological analysis of intestines of Jian carp juvenile exposed to polystyrene microspheres. Scale bar = 50 μm.


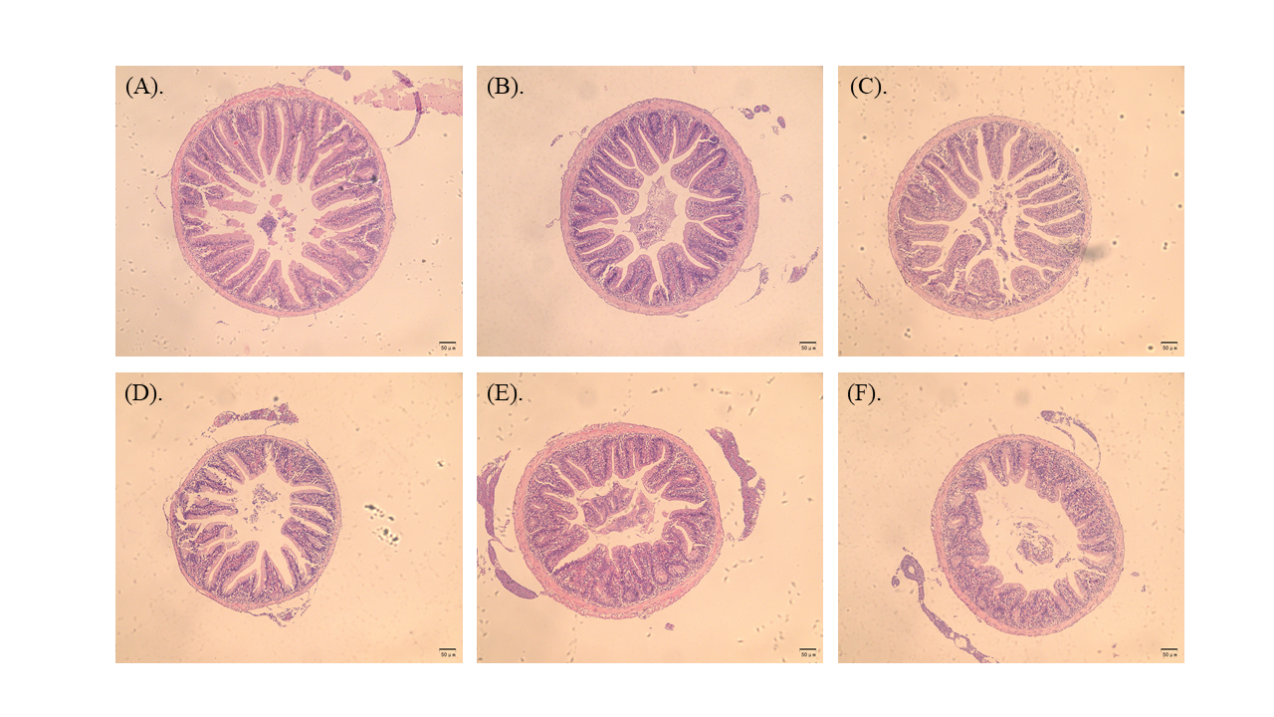


**Supplementary table 1. Intestinal muscular thickness and intestinal** **villi length of Jian carp**

| **Size** | **Concentration** | **Intestinal villi length (μm)** | | **Intestinal muscular thickness (μm)** | |
| --- | --- | --- | --- | --- | --- |
|  |  | E48h | C48h | E48h | C48h |
| 80 nm | 0 | 168.46±50.18 | 136.94±34.17 | 23.78±5.66 | 25.16±3.3 |
|  | 20 μg/L | 201.27±28.03 | 122.06±25.93 | 36.62±6.52 | 27.19±11.18 |
|  | 200 μg/L | 135.18±22.49 | 133.61±18.71 | 21.52±6.51 | 29.82±4.16 |
|  | 2000 μg/L | 146.47±26.32 | 152.21±35.87 | 34.59±9.98 | 33.66±6.45 |
| 8 μm | 0 | 160.9±28.72 | 115.58±19.62 | 22.49±5.22 | 26.39±5.97 |
|  | 20 μg/L | 139.89±33.24 | 142.07±27.9 | 28.12±7.08 | 35.2±7.92 |
|  | 200 μg/L | 109.02±35.3 | 141.54±42.61 | 25.83±6.84 | 30.41±9.67 |
|  | 2000 μg/L | 138.47±39.92 | 116.32±16.04 | 30.63±5.68 | 27.12±0.92 |

**Supplement Text 1.** **Details of experimental methods of RNA extraction and cDNA synthesis.**

Total RNA was extracted using AG RNAex Pro Rreagent (Accurate Biotechnology Co., Ltd, Hunan, China) following the manufacturer’s protocol. RNA quality was assessed by electrophoresis on a 1.0% agarose gel and its concentration was tested by mySPEC (VWR, Radnor, PA, USA). 1 μg total RNA was purified and the first-strand cDNA was synthesized using Evo M-ML RT Kit with gDNA Clean for qPCR (Accurate Biotechnology Co., Ltd, Hunan, China) according to the manufacturer’s instructions.

**Supplement Text 2. Details of the qPCR program.**

The real-time PCR program was set at 30 s for 95 ℃, followed by 40 cycles of 95℃ for 5s, 60℃ for 30s. Melting curves were obtained by increasing the temperature from 60 to 95℃ (0.5℃/s) to denature the double-stranded DNA. Each amplification reaction was run in triplicate. After finishing the program, the threshold cycle (Ct) values were obtained from each sample. Relative gene expression levels were evaluated using 2^−ΔΔCT^ method.
